# Thermally Activated Delayed Fluorescence Material: An Emerging Class of Metal‐Free Luminophores for Biomedical Applications

**DOI:** 10.1002/advs.202102970

**Published:** 2021-10-27

**Authors:** Fang Fang, Lin Zhu, Min Li, Yueyue Song, Meng Sun, Dongxu Zhao, Jinfeng Zhang

**Affiliations:** ^1^ Key Laboratory of Molecular Medicine and Biotherapy, School of Life Sciences Beijing Institute of Technology Beijing 100081 P. R. China

**Keywords:** biosensing, metal‐free luminescent materials, photodynamic therapy (PDT), time‐resolved luminescence imaging (TRLI), thermally activated delayed fluorescence (TADF)

## Abstract

The development of simple, efficient, and biocompatible organic luminescent molecules is of great significance to the clinical transformation of biomaterials. In recent years, purely organic thermally activated delayed fluorescence (TADF) materials with an extremely small single‐triplet energy gap (Δ*E*_ST_) have been considered as the most promising new‐generation electroluminescence emitters, which is an enormous breakthrough in organic optoelectronics. By merits of the unique photophysical properties, high structure flexibility, and reduced health risks, such metal‐free TADF luminophores have attracted tremendous attention in biomedical fields, including conventional fluorescence imaging, time‐resolved imaging and sensing, and photodynamic therapy. However, there is currently no systematic summary of the TADF materials for biomedical applications, which is presented in this review. Besides a brief introduction of the major developments of TADF material, the typical TADF mechanisms and fundamental principles on design strategies of TADF molecules and nanomaterials are subsequently described. Importantly, a specific emphasis is placed on the discussion of TADF materials for various biomedical applications. Finally, the authors make a forecast of the remaining challenges and future developments. This review provides insightful perspectives and clear prospects towards the rapid development of TADF materials in biomedicine, which will be highly valuable to exploit new luminescent materials.

## Graphical abstract

The review focuses on summarizing the current progresses of TADF materials for various biomedical applications, including conventional fluorescence imaging, time‐resolved imaging and sensing, and photodynamic therapy. Moreover, the TADF mechanisms and design strategies of TADF fluorophores in biomedicine are also described. Finally, it provides the insightful perspectives and clear prospects towards the rapid development of TADF materials in biomedicine.

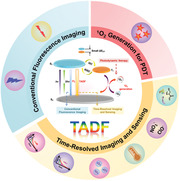

## 1 Introduction

**Figure 1:**
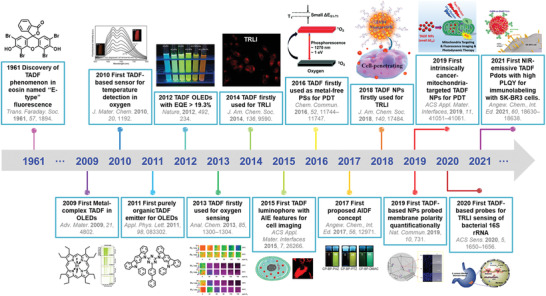
Timeline of the major developments of TADF materials for different applications including optoelectronics, bioimaging, biosensors, and nanomedicine. (EQE: external quantum efficiency; TRLI: time‐resolved luminescence imaging; AIE: aggregation‐induced emission; PSs: photosensitizers; PDT: photodynamic therapy; AIDF: aggregation‐induced delayed fluorescence; NPs: nanoparticles; PLQY: photoluminescence quantum yield.) Left to right: Reproduced with permission.^[^ ^18^ ^]^ Copyright 1961, The Royal Society of Chemistry. Reproduced with permission.^[^ ^19^ ^]^ Copyright 2009, WILEY‐VCH. Reproduced with permission.^[^ ^20^ ^]^ Copyright 2010, The Royal Society of Chemistry. Reproduced with permission.^[^ ^21^ ^]^ Copyright 2011, American Institute of Physics. Reproduced with permission.^[^ ^7^ ^]^ Copyright 2012, Nature Publishing Group. Reproduced with permission.^[^ ^22^ ^]^ Copyright 2013, American Chemical Society. Reproduced with permission.^[^ ^23^ ^]^ Copyright 2014, American Chemical Society. Reproduced with permission.^[^ ^24^ ^]^ Copyright 2015, American Chemical Society. Reproduced with permission.^[^ ^25^ ^]^ Copyright 2016, The Royal Society of Chemistry. Reproduced with permission.^[^ ^26^ ^]^ Copyright 2017, WILEY‐VCH. Reproduced with permission.^[^ ^27^ ^]^ Copyright 2018, American Chemical Society. Reproduced with permission.^[^ ^28^ ^]^ Copyright 2019, Nature Publishing Group. Reproduced with permission.^[^ ^14^ ^]^ Copyright 2019, American Chemical Society. Reproduced with permission.^[^ ^15^ ^]^ Copyright 2020, American Chemical Society. Reproduced with permission.^[^ ^17^ ^]^ Copyright 2021, WILEY‐VCH.

Over the past decades, the rapid developments of both materials technology and biomedicine have accelerated the exploitation of a wide variety of new functional materials, providing potential alternatives for diagnosis and treatment of various diseases, especially cancer.^[^ ^1^, ^2^, ^3^, ^4^, ^5^, ^6^ ^]^ However, exploring advanced luminescent materials as theranostic agents with ideal photophysical properties and reliable biosafety is still urgently needed. In recent years, a report of thermally activated delayed fluorescence (TADF) materials with an extremely small singlet‐triplet energy gap (Δ*E*_ST_) performing theoretically 100% internal quantum efficiencies (QE) via the reverse intersystem‐crossing (RISC) process, has been regarded as a significant breakthrough in the field of organic light‐emitting diodes (OLEDs).^[^ ^7^, ^8^, ^9^, ^10^, ^11^ ^]^ Since the pioneering Nature paper of Adachi and co‐workers in 2012, considerable efforts have been triggered to develop efficient TADF materials for different applications including optoelectronics, bioimaging, biosensors, and nanomedicine (**Figure** **1**). Strikingly, by virtue of the tailorable synthesis, low‐cost input, favorable photophysical features (e.g., long‐lived emissive feature), and excellent biocompatibilities (e.g., metal‐free molecular structure), the purely organic TADF materials have shown fascinating appeal in the field of biomedicine.^[^ ^12^, ^13^, ^14^, ^15^, ^16^, ^17^ ^]^

**Figure 2:**
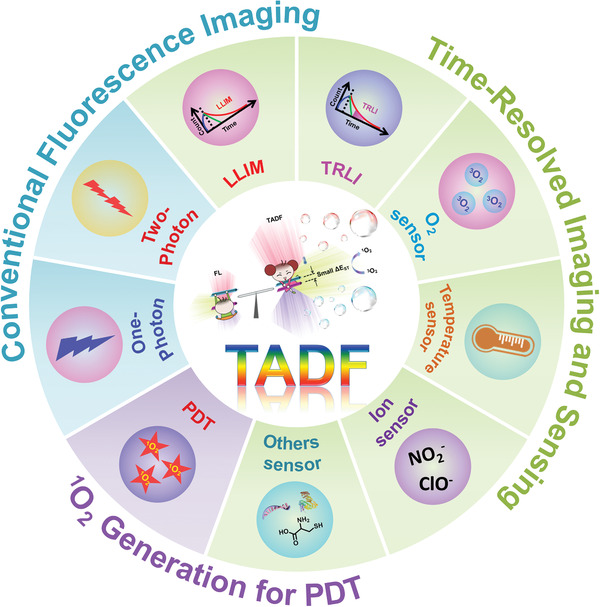
Schematic illustration of TADF materials for different biomedical applications, including conventional fluorescence imaging, time‐resolved imaging and sensing, as well as PDT.

Up to now, substantial contributions have been made to develop TADF materials for biomedical applications. First, as typical metal‐free luminophores, TADF emitters could be utilized for conventional fluorescence imaging in different biological systems.^[^ ^24^, ^29^ ^]^ Meanwhile, the long‐lived emission feature renders the TADF materials fluorescence lifetime imaging (FLIM) or time‐resolved luminescence imaging (TRLI) capability, which could eliminate the interference of background signals and improve the accuracy in bioimaging.^[^ ^12^, ^27^, ^30^ ^]^ Moreover, the TADF phenomenon originates from the RISC process, which is highly sensitive to both thermal energy and oxygen concentration, such characters could be applied to temperature and oxygen sensing.^[^ ^31^, ^32^ ^]^ Beyond these, the TADF molecules with small Δ*E*_ST_ and efficient intersystem‐crossing (ISC) process could also be used as novel metal‐free photosensitizers (PSs) for photodynamic therapy (PDT). This is because that along with the excited triplet state (T_1_) decays back to the excited singlet state (S_1_), energy could be transferred to the surrounding oxygen leading to the reactive singlet oxygen (^1^O_2_) generation for PDT.^[^ ^14^, ^16^, ^25^, ^33^ ^]^ In brief, such inherent photophysical properties of TADF molecules could be delicately tailored through manipulating the molecular structures for different biomedical applications, such as bioimaging, sensing, and PDT, which will be explained in detail below (**Figure** **2**).

Although TADF materials have shown attractive potential in biomedicine, their development is still in its infancy, lacking systematic summary and prospect of the current progress in TADF molecules. In this review, we will first introduce the molecular mechanisms of TADF materials in biomedicine. Subsequently, the synthesis principles of TADF molecules as well as design strategies of TADF nanomaterials will be discussed. Afterwards, special focus will be placed on the biomedical applications of TADF luminophores, including conventional fluorescence imaging, time‐resolved imaging and sensing, as well as PDT. Finally, some insightful perspectives will be provided to highlight the remaining challenges and possibilities of the currently developed TADF materials in biomedicine, which will be beneficial to the future design and development of luminescent materials.

## 2 Mechanisms of TADF Materials for Biomedical Applications

**Figure 3:**
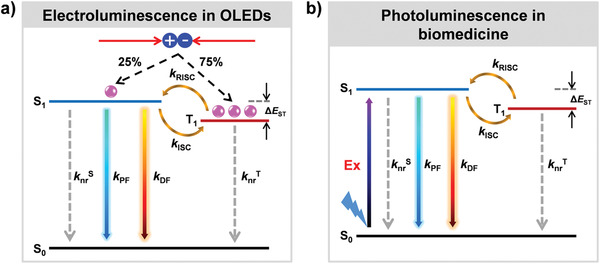
Simplified schematic representation of the TADF processes by a) electroexcitation for OLEDs or b) photoexcitation for biomedical applications. (*k*_nr_^S^ and *k*_nr_^T^: the non‐radiative decay constants of S_1_ and T_1_, respectively; *k*_PF_ and *k*_DF_: the rate constants of prompt fluorescence (PF) and delayed fluorescence (DF) processes, respectively; *k*_ISC_ and *k*_RISC_: the intersystem crossing (ISC) and reversible intersystem crossing (RISC) rate constants, respectively. Phosphorescence is ignored.)

Typically, the distinct optical and electronic properties of TADF materials could be attributed to the sufficiently small Δ*E*_ST_ between the S_1_ and T_1_ states, enabling an efficient RISC process and a resultant ≈100% internal QE by harvesting both singlet and triplet excitons, which is a crucial theoretical breakthrough in organic electronics.^[^ ^34^, ^35^, ^36^, ^37^, ^38^, ^39^, ^40^ ^]^ Likewise, such unique optical properties render TADF materials as promising candidates for biomedical applications. Generally, two types of luminescence mechanisms are represented in TADF: the prompt fluorescence (PF) and the delayed fluorescence (DF), which could be triggered by photoexcitation or electroexcitation.^[^ ^41^, ^42^, ^43^, ^44^ ^]^ In light‐emitting devices such as OLEDs, the TADF emitters are electronic excitation (**Figure** **3**a), where 25% singlet and 75% triplet excitons are generated via hole and electron injection. Assuming non‐radiative decay and phosphorescence emission are ignored, the triplet excitons could retransform into singlet excitons by RISC process, and both the initial and back‐formed singlet excitons could realize PF and DF emission. Hence, the efficient triplet to singlet transformation is essential to maximize electroluminescence of TADF devices for OLEDs applications. On the other hand, in various biomedical applications, the TADF fluorophores are optically excited (Figure 3b), where only singlet excitons are formed and triplet excitons are generated through the ISC process, and the PF and DF with different fluorescent lifetimes could also be observed, which would be described in detail following.^[^ ^8^, ^45^ ^]^ Of particular note, some recent studies have elaborated that in many TADF materials, especially the donor–acceptor (D–A) and donor‐acceptor‐donor (D–A–D) type TADF molecules which possess strong intramolecular charge‐transfer (CT), the spin‐orbit coupling (SOC) between the ^1^CT and ^3^CT states is forbidden when the exchange energy between the near orthogonal D and A units is almost zero. In the scenario, a more complex second‐order spin‐vibronic coupling mechanism enables us to further understand the TADF dynamical mechanisms, where an energetically nearest local triplet (^3^LE) state is as an intermediary state coupling ^3^CT and ^1^CT states and thus inducing the second‐order SOC.^[^ ^46^, ^47^, ^48^, ^49^, ^50^, ^51^ ^]^

**Figure 4:**
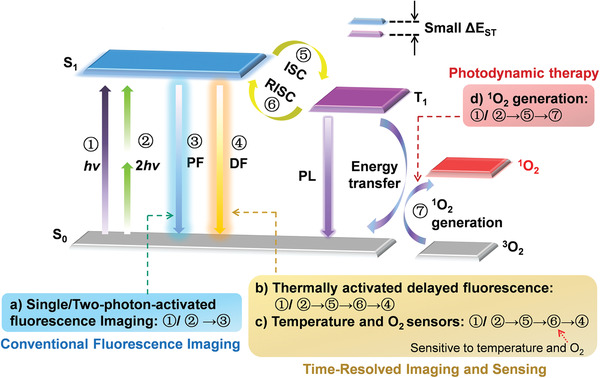
Simplified diagram illustrating molecular mechanisms of TADF materials for biomedical applications: a) conventional fluorescence imaging; b,c) time‐resolved luminescence imaging and sensing; d) ^1^O_2_ generation for PDT. (①: single‐photon (*hv*) excitation, ②: two‐photon (2*hv*) excitation, ③: prompt fluorescence (PF) emission, ④: delayed fluorescence (DF) emission, ⑤: intersystem crossing (ISC) process, ⑥: reversible intersystem crossing (RISC) process, ⑦ energy transfer and ^1^O_2_ generation, and ⑧: phosphorescence (Phos) emission (Not emphasized in this review).

In this review, we will focus on the detailed mechanisms of TADF materials in biomedicine, as elaborated in **Figure** **4**. For bioimaging, When the TADF materials are excited by single photon or two photons, they will get activated from the ground state (S_0_) to the excited S_1_ state. On one hand, the excited TADF molecules will experience a fast radiative decay back to S_0_ by emitting PF, showing a short lifetime (typically less than 10 ns) for conventional fluorescence imaging (Figure 4a). Alternatively, the excited TADF molecules will undergo an ISC process from the S_1_ state to the T_1_ state. Subsequently, the small Δ*E*_ST_ will facilitate a thermally activated up‐conversion from non‐emissive triplet excitons into spin‐allowed singlet excitons by an efficient RISC process, thus leading to the DF emission, which is the key to the TADF mechanism (Figure 4b).^[^ ^8^, ^45^, ^52^ ^]^ Obviously, TADF molecules display a sufficiently longer fluorescence lifetime ranging from microseconds to milliseconds due to the population of the excited S_1_ state originating from the T_1_ state. In comparison to the short‐lived PF hardly distinguished from the background signals within nanosecond range in biological environments,^[^ ^53^ ^]^ the DF with much longer lifetime could be detected over a wider timescale, which is highly desirable for time‐resolved imaging such as FLIM and TRLI, showing high signal‐to‐noise ratio, remarkable sensitivity, and superior temporal‐spatial resolution in bioimaging.^[^ ^42^, ^54^, ^55^ ^]^

Of particular note, the TADF phenomenon‐involved RISC process is extremely sensitive to both the oxygenic environment and thermal energy. For the former, oxygen‐induced triplet quenching effect can be regarded as a “double‐edged sword”. On one hand, it would severely hinder the DF‐based FLIM and TRLI applications, on the other hand, it could be utilized as efficient optical sensors for molecular oxygen in various biological mediums (Figure 4c).^[^ ^14^, ^22^, ^25^, ^56^, ^57^ ^]^ Moreover, because the thermally activated RISC in TADF emitters is also susceptible to temperature, it could be applied to temperature detection within a broad range from −75 to 105 °C.^[^ ^8^ ^]^ The relationship between the RISC rate constant (*k*_RISC_) and temperature could be estimated from the Boltzmann distribution relation (Equation (1)):

(1) $$k_{\mathrm{RISC}}\propto \exp\left(\frac{\Delta E_{\mathrm{ST}}}{k_{B}T}\right)$$

where *T* is the temperature and *k*_B_ is the Boltzmann constant.^[^ ^8^, ^43^ ^]^ It should be noted that although the higher temperature would facilitate the *k*_RISC_, the photoluminescence quantum yield (PLQY) of DF may decrease because of the concurrently increased non‐radiative deactivation, so the strongest TADF signal could be realized through optimizing temperature. Meanwhile, the Arrhenius model (Equation (2)) reveals that the fluorescence lifetime (*τ*) is a function of temperature (*T*):

(2) $$\tau ={\left(k_{0}+k_{1}e^{-\frac{\Delta E_{\mathrm{ST}}}{k_{B}T}}\right)}^{-1}$$

where *k*_1_ is the pre‐exponential factor and *k*_0_ is the temperature‐independent decay rate.^[^ ^42^ ^]^ It demonstrates that the change of lifetime could be measured to detect the temperature of environments (Figure 4c).

Apart from imaging and sensing applications involving TADF phenomena, the extremely small Δ*E*_ST_ and the resultant highly‐efficient ISC process endow the purely organic TADF emitters with robust photosensitization, which could be served as promising PSs for PDT. As described in Figure 4d, similar with typical PSs, when TADF emitters excited to a short‐lived S_1_ state upon photo‐irradiation, the formed S_1_ excitons undergo ISC to a long‐lived T_1_ state, and subsequently transfer energy to the surrounding ^3^O_2_, leading to the formation of cytotoxic ^1^O_2_ for PDT application.^[^ ^58^, ^59^ ^]^ Previous studies have shown that the ISC process is pivotal to achieve high‐efficient reactive oxygen sensitization.^[^ ^25^, ^60^, ^61^, ^62^, ^63^, ^64^ ^]^ According to Fermi's Golden rule, the dependence of the ISC rate constant (*k*_ISC_) on spin–orbit coupling (SOC) and Δ*E*_ST_ could be evaluated from Equation (3):

(3) $$k_{\mathrm{ISC}}\propto \frac{{\left(T_{1}\left|H_{\mathrm{SO}}\right|S_{1}\right)}^{2}}{\Delta {E_{\mathrm{ST}}}^{2}}$$

where H_SO_ is the Hamiltonian for SOC. In comparison to introducing the heavy atoms (e.g., Gd, Ir, Ru, etc.) into PS molecules to increase H_SO_ for improving *k*_ISC_ and ^1^O_2_ yield,^[^ ^6^, ^9^, ^10^, ^11^, ^65^ ^]^ the metal‐free TADF materials with small Δ*E*_ST_ enabling much strong ISC process and efficient PDT performance, display much lower cytotoxicity, and complete biodegradability. In this regard, the TADF emitters containing no heavy metals substantially reduce concerns about toxicity, non‐degradation, and manufacturing cost issues. It is also worthwhile to note that the above‐mentioned oxygen‐induced triplet quenching effect of TADF materials could be taken advantage for ^1^O_2_ generation in PDT by preventing the RISC process and hence retaining the TADF molecule in the excited T_1_ state.

## 3 Design Strategies of TADF Materials in Biomedicine

Although abundant TADF molecules including carbazole derivatives, fluorescein derivatives, and anthraquinone derivatives have been elaborately synthesized for the fabrication of optoelectronic devices, reliable TADF luminophores with ideal photophysical properties and satisfactory biological functions for specific biomedical applications are still urgently needed. Furthermore, poor aqueous solubilities, undesired optical performance in polar media, and emission quenching by surrounding molecular oxygen are other intractable issues limiting the applications of TADF compounds in biomedicine.^[^ ^56^, ^57^, ^66^, ^67^, ^68^ ^]^ So far, several representative design strategies have been established to solve the above dilemma towards TADF materials in biomedicine (**Table** **1**). In general, by means of molecular design and delicate synthesis such as integration of AIE features,^[^ ^66^, ^69^, ^70^ ^]^ modification with hydrophilic or targeting moieties,^[^ ^15^, ^30^, ^69^ ^]^ as well as copolymerization with other fluorophores or functional molecules,^[^ ^71^ ^]^ numerous TADF molecules have been equipped with additional properties. Meanwhile, the fabrication of TADF luminophore‐based nanomaterials has also been regarded as an appealing strategy to extend the bio‐application of TADF materials, including direct self‐assembly of TADF molecules,^[^ ^14^, ^24^, ^25^, ^29^ ^]^ encapsulation TADF molecules within polymers,^[^ ^12^, ^13^, ^16^, ^47^, ^72^, ^73^ ^]^ peptides,^[^ ^27^ ^]^ or other nanocarriers,^[^ ^41^, ^67^, ^68^, ^74^ ^]^ self‐assembly^[^ ^66^ ^]^ or reverse microemulsion^[^ ^75^ ^]^ of the modified TADF molecules, and copolymerizing TADF monomers with host materials for further coprecipitation.^[^ ^32^ ^]^

**Table 1:** Design strategies of TADF materials for biomedical applications

Design strategies	Types	Advantages	TADF molecules	Auxiliary components	Biomedical applications	Refs.
Integration of TADF and AIE properties into one molecule named AIDF	Molecular scale	Versatile, flexible, alleviates the ACQ and O_2_ quenching effects, improves the photophysical property	PXZT	/	TRLI	^[^ ^76^ ^]^
			PXZ‐NI, PTZ‐NI, Lyso‐PXZ‐NI	/	TRLI	^[^ ^70^ ^]^
Modification by hydrophilic or targeting moieties	Molecular scale	Universal, improves water dispersibility, enhances targeting ability	NID	A TPP^+^ group	Mitochondrion‐targeted TRLI	^[^ ^69^ ^]^
			AI−Cz−CA	5″‐NH_2_ neomycin	Dual‐mode detection of bacterial 16S rRNA in tissues	^[^ ^15^ ^]^
			AI‐Cz‐CA	Triphenylphosphonium (TPP), 2‐morpholinoethylamine	TRLI	^[^ ^30^ ^]^
Copolymerization with other monomers	Molecular scale	Incorporates different fluorophores or functional molecules into a TADF‐based polymer	NAI‐DMAC, NAI‐PTZ, NAI‐POZ	*t*BuODA, NIPAM	Ratiometric temperature sensing	^[^ ^71^ ^]^
Self‐assembly	Nanoscale	Simple, low‐cost, reproducible, alleviates the sensitivity to oxygen	TPAAQ	/	Long‐term cellular imaging	^[^ ^24^ ^]^
			2CzPN, 4CzIPN, 4CzTPN‐Ph	/	One/two‐photon cellular imaging	^[^ ^29^ ^]^
			2CzPN, 4CzIPN, 4CzTPN‐Ph	/	The first report of TADF applied for PDT	^[^ ^25^ ^]^
			An‐TPA, An‐Cz‐Ph	/	Mitochondrion‐targeted two‐photon imaging and PDT	^[^ ^14^ ^]^
Encapsulation within different nanocarriers	Nanoscale	Universal, increases the bioavailability and water solubility, enhances blood circulation time and local accumulation	TXO	PEG‐*b*‐PPG‐*b*‐PEG	Two‐photon FLIM	^[^ ^73^ ^]^
			4CzIPN	PEG‐*b*‐PPG‐*b*‐PEG	Two‐photon FLIM	^[^ ^53^ ^]^
			CPy	DSPE‐PEG2000	TRLI in cells and in vivo	^[^ ^72^ ^]^
			4CzIPN, NAI‐DPAC, BTZ‐DMAC	Amphiphilic cell‐penetrating peptide, F_6_G_6_(rR)_3_R_2_	TRLI	^[^ ^27^ ^]^
			M‐1	Pluronic F‐127	TRLI	^[^ ^12^ ^]^
			DCzB	Amphiphilic phospholipid F127	Afterglow cell imaging and visual temperature detection	^[^ ^13^ ^]^
			PT, AT	DSPE‐PEG2000	Two‐photon nanotheranostics	^[^ ^16^ ^]^
			4CzIPN	Host matrices mCP and mCBP, DSPE‐PEG2000	TRLI	^[^ ^68^ ^]^
			DPTZ‐DBTO_2_, TXO‐TPA	Polystyrene (PS) NPs	Fluorescence cell imaging	^[^ ^41^ ^]^
			TADF	Galactose‐PEG‐DSPE modified liposome	Improved malignant cells uptake for fluorescence imaging	^[^ ^74^ ^]^
			BP‐2PTZ, BP‐PXZ, BP‐2PXZ, BP‐PTZ	BSA	Fluorescence imaging and FLIM	^[^ ^67^ ^]^
Self‐assembly or reverse microemulsion of the modified TADF molecules	Nanoscale	Enhances targeting ability and reduces triplet quenching by oxygen	AI‐Cz	A hydrophilic chain	Fluorescence imaging and FLIM	^[^ ^66^ ^]^
			DCF‐BYT	APTES	Single photon imaging	^[^ ^75^ ^]^
Coprecipitation of the host material‐copolymerized TADF with amphiphilic polymers	Nanoscale	Obtains multifunctional TADF materials	Acrylic oxadiazole TADF monomer	CzBA‐based host materials, polystyrene/maleic anhydride copolymers	Ratiometric O_2_ sensing	^[^ ^32^ ^]^

### 3.1 Design Principles of TADF Molecules

**Figure 5:**
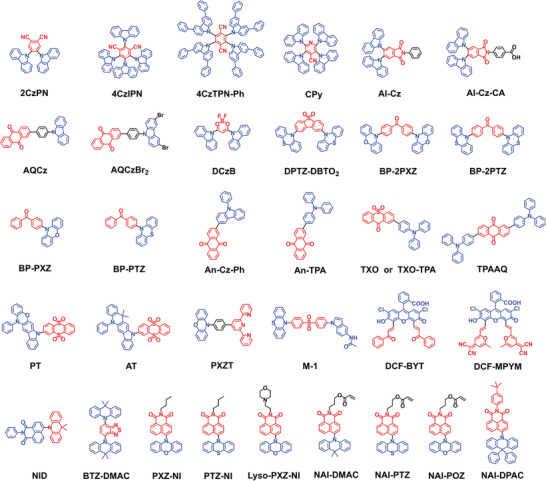
Typical TADF molecular structures rationally designed for different biomedical applications.

Typically, a satisfying TADF molecule requires an extremely small Δ*E*_ST_ of ≤100 meV, which will render the efficient RISC. Such small Δ*E*_ST_ could be attainable by designing a TADF molecular with distinct electron donor (D) and acceptor (A) segments through the twisted intramolecular charge transfer (TICT) strategy to reduce the overlap between the highest occupied molecular orbital (HOMO) and the lowest unoccupied molecular orbital (LUMO), ultimately minimizing the exchange interactions and achieving the TADF characteristics. In comparison with the TADF molecules with relatively larger Δ*E*_ST_ which are favorable for fluorescence emission, those with the extremely small Δ*E*_ST_ tend to transfer energy from the T_1_ state to the surrounding oxygen for the ROS formation.^[^ ^13^, ^16^ ^]^ Therefore, the molecular structure of the TADF luminophores could be rationally designed according to specific biological applications. Typical TADF molecular structures synthesized for different biomedical applications is shown in **Figure** **5**. Furthermore, the through‐space charge‐transfer (TSCT) effect and multiple‐resonance (MR) effect are also general molecule design strategies to develop TADF materials in OLEDs: the former can be used to fabricate TADF polymers based on a nonconjugated polymer backbone and spatially separated D and A units;^[^ ^77^, ^78^ ^]^ and the latter can be utilized for construction of TADF compounds with small Δ*E*_ST_ (≈200 meV), narrowband emission, and high PLQY.^[^ ^79^, ^80^, ^81^ ^]^ To the best of our knowledge, these two approaches have not yet been applied to prepare TADF dyes in biomedicine.

In addition, similar to conventional organic fluorescent molecules, most TADF molecules also suffer from the notorious aggregation‐caused quenching (ACQ) effect, making their bioimaging application more challenging. Fortunately, with the advancement of aggregation‐induced emission (AIE) field, an effective strategy of integrating the TADF features with AIE characteristics in a single molecule termed aggregation‐induced delayed fluorescence (AIDF) is developed, which not only restrains the non‐radiative transition but also secludes TADF molecules from the surrounding oxygen, shedding a light on the development of TADF materials with favorable emission efficiency for bioimaging.^[^ ^16^, ^24^, ^42^, ^56^, ^60^, ^66^, ^67^, ^68^, ^69^, ^70^, ^73^, ^76^ ^]^ As the forerunner, Tang's group synthesized a series of AIDF luminogens (BP‐2PXZ, BP‐PXZ, BP‐2PTZ, and BP‐PTZ) through integrating AIE and TADF properties within one molecule, showing a strong and long‐lived fluorescence emission in aggregated states.^[^ ^67^ ^]^ Moreover, Yoon et al. designed TADF molecules based on electron donor (PXZ or PTZ as donor) and electron acceptor (1,8‐naphthalimide, NI), which exhibited prominent aggregation‐induced red‐emission and prolonged fluorescence lifetime.^[^ ^70^ ^]^

On the other hand, covalent or non‐covalent modifications of the TADF luminophores with hydrophilic groups or targeting ligands as effective strategies to respectively endow these molecules with water dispersibility or targeting capability.^[^ ^15^, ^30^, ^69^ ^]^ In 2019, Yang et al. introduced the triphenylphosphonium (TPP^+^) group to the hydrophobic TADF emitter 6‐(9,9‐dimethylacridin‐10(9H)‐yl)‐2‐phenyl‐1H‐benzo[de]isoquinoline‐1,3(2H)‐dione (NID) via nonconjugated linking, ultimately forming the hydrophilic NID‐TPP, which displayed obvious aggregation‐induced delayed fluorescence enhancement (AIDFE) under the specific accumulation in mitochondria.^[^ ^69^ ^]^ Very recently, Hu’ group conjugated the TADF luminophore AI−Cz−CA with the hydrophilic neomycin, which could specifically targeting the bacterial 16S ribosomal RNA, exhibiting long‐lived delayed fluorescence in oxygen‐containing environments. Furthermore, polymerization, for example, Cu(0)‐reversible deactivation radical polymerization (Cu(0)‐RDRP), was applied to construct TADF‐based copolymers, which could integrate different luminophore monomers to expand the optical applications.^[^ ^71^ ^]^

### 3.2 Design Strategies of TADF Nanomaterials

Although obvious progress has been made to improve the water dispersibility and targeting ability of TADF molecules via chemical structure modification, there is still considerable room to further address those challenges severely restricting the practical bio‐applications of TADF compounds. The booming development of nanotechnology has provided a powerful tool to minimize the impediments to TADF molecules including intrinsic hydrophobicity and emission quenching by surrounding O_2_ or physiological aqueous environment. In recent years, fabrication of diverse TADF nanoplatforms has emerged as the most robust methodology for employing the TADF luminophores.^[^ ^14^, ^16^, ^24^, ^27^, ^41^, ^68^, ^82^, ^83^ ^]^ In this way, the rotational and vibrational degrees of freedom in TADF molecules could be suppressed when encapsulated within NPs, resulting in enhancing their luminescence performance in biomedical research.^[^ ^42^ ^]^

**Figure 6:**
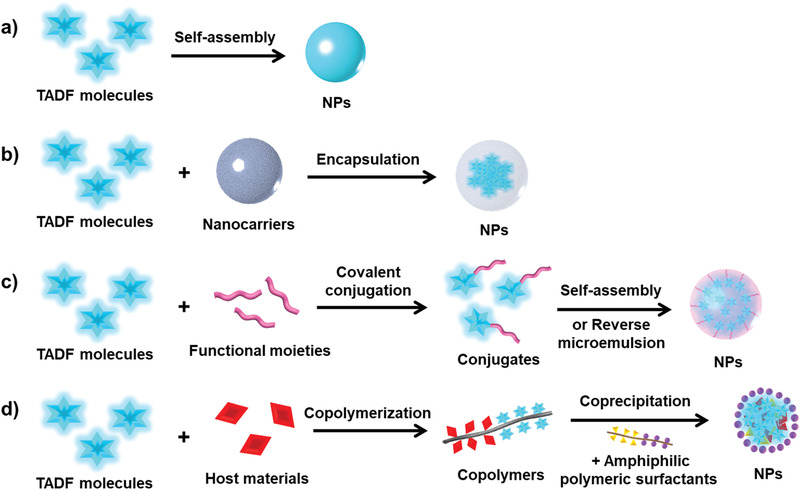
Schematic illustration of design strategies of the TADF nanomaterials in biomedicine: a) direct self‐assembly of unmodified TADF molecules, b) encapsulation of the TADF molecules within different nanocarriers, c) self‐assembly or reverse microemulsion of the modified TADF molecules, and d) coprecipitation of an amphiphilic polymeric surfactant with the host material‐copolymerized TADF.

As depicted in **Figure** **6**, four representative strategies have been developed to prepare TADF nanomaterials in biomedicine. First, through direct supramolecular self‐assembly, one of the most widely applied and reproducible method for preparing NPs,^[^ ^84^, ^85^, ^86^, ^87^ ^]^ our group has fabricated a series of TADF nanomaterials based on anthraquinone derivatives^[^ ^14^, ^24^ ^]^ or carbazole derivatives,^[^ ^25^, ^29^ ^]^ which revealed the tailorable synthesis, excellent photoproperties, and superb image‐guided PDT theranostic capability (Figure 6a). Another popular strategy lies on encapsulating the TADF molecules into different nanocarriers such as polymers,^[^ ^12^, ^13^, ^16^, ^53^, ^72^, ^73^ ^]^ peptides,^[^ ^27^ ^]^ liposomes,^[^ ^74^ ^]^ bovine serum albumin (BSA),^[^ ^67^ ^]^ polystyrene NPs,^[^ ^41^ ^]^ and glassy organic host matrix,^[^ ^68^ ^]^ isolating them from adverse oxygenic and aqueous environments for live‐cell fluorescence imaging, FLIM, or TRLI (Figure 6b). For instance, Fan et al. constructed water‐dispersible organic semiconducting nanoparticles (TXO NPs) by trapping the TADF fluorophore within an amphiphilic copolymer (PEG‐*b*‐PPG‐*b*‐PEG), which could achieve long‐lived fluorescence lifetime and efficient two‐photon absorption (TPA) for in vivo FLIM.^[^ ^73^ ^]^ In addition, the lipophilic TADF molecules could also be covalently anchored with hydrophilic chain to form an amphiphilic TADF monomer, which could be further fabricated into NPs with good water dispersibility, excellent stability, and well‐preserved long TADF lifetime via self‐assembly or reverse microemulsion (Figure 6c).^[^ ^66^, ^75^ ^]^ Furthermore, there is a recent report on copolymerization of the synthesized TADF monomers with a suitable host materials by Cu(0)‐RDRP, the resultant copolymers were subsequently formed into NPs to improve the performance of TADF materials in ratiometric oxygen sensing (Figure 6d).^[^ ^32^ ^]^

Despite molecular structure modification and encapsulating TADF compounds within nanomaterials are two effective strategies to improve the intrinsic drawbacks of TADF molecules as well as protect the long‐lived T_1_ states from unfavorable nonradiative relaxations, only limited TADF compounds have been successfully developed in biomedical research. Therefore, more effort should be devoted to exploit highly efficient TADF materials for boosting their biomedical outcomes.

## 4 TADF Materials for Biomedical Applications

### 4.1 Conventional Fluorescence Imaging

**Figure 7:**
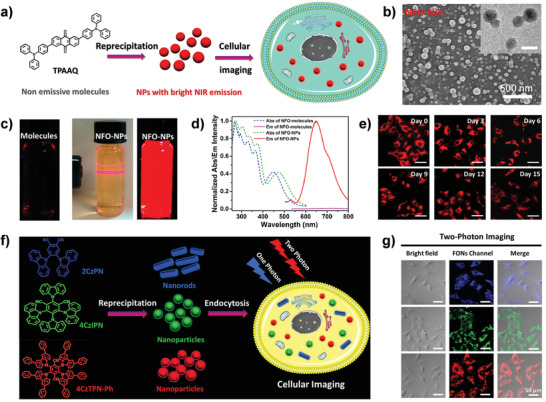
TADF materials for conventional fluorescence imaging. a) Scheme of fabrication of the NFO‐NPs for cellular imaging. b) SEM image of the NFO‐NPs. c) Photographs of TPAAQ in THF under UV light, and the NFO‐NPs in water under room light or UV light. d) Normalized absorbance and fluorescence spectra of TPAAQ in THF and the NFO‐NPs in deionized water. e) Long‐term cellular fluorescence images of the NFO‐NPs. a‐e) Reproduced with permission.^[^ ^24^ ^]^ Copyright 2015, American Chemical Society. f) The construction of the blue/green/red FONs for one‐photon/two‐photon fluorescence imaging. g) One‐photon/two‐photon cellular tracing of the three FONs. f,g) Reproduced with permission.^[^ ^29^ ^]^ Copyright 2016, American Chemical Society.

Fluorescence imaging is a versatile and indispensable tool in biomedical research and clinical applications, exhibiting ultrahigh sensitivity and superior temporal‐spatial resolution.^[^ ^88^, ^89^, ^90^, ^91^, ^92^, ^93^ ^]^ Compared with the traditional fluorophores such as inorganic semiconductors and fluorescent polymers, purely organic TADF dyes possess desirable PLQY, delicate structure tailoring, favorable biodegradability, and low toxicity, demonstrating promising potential for bioimaging over the past few years.^[^ ^24^, ^29^, ^68^ ^]^ For instance, our group designed TADF nanoprobes (NFO‐NPs) based on anthraquinone derivate (TPAAQ) via simple self‐assembly (**Figure** **7** **a,b**). As exhibited in Figure 7c, contrary to TPAAQ molecules in THF with negligible fluorescence, the as‐prepared NPs displayed strong red emission, confirming the AIE features of NFO‐NPs. Besides, the large Stokes shift (177 nm) revealed its potential for bioimaging with remarkable signal‐to‐noise ratio (Figure 7d). Afterwards, long‐term cellular tracing capability of the NFO‐NPs has also been determined where their red fluorescence signal could still be observed after 15 days upon adding NPs only once (Figure 7e).^[^ ^24^ ^]^ Moreover, Adachi et al. fabricated photostable and long‐lived TADF O‐dots through loading 2,4,5,6‐tetrakis(carbazol‐9‐yl)‐1,3‐dicyanobenzene (4CzIPN) luminophores into a glassy host matrix, exhibiting a more remarkable PLQY (94%) compared to the pure 4CzIPN O‐dots (13%) in aqueous solution, which is encouragingly promising for bioimaging both in vitro and in vivo.^[^ ^68^ ^]^

In addition to poor water solubility and oxygen sensitivity, another major challenge of TADF emitters in bioimaging is the short‐wavelength excitations, which might not only damage cells or tissues but also result in the adverse background signals of autofluorescence and poor optical penetration depth. To eliminate these shortcomings, our group used carbazole derivatives (2CzPN, 4CzIPN, 4CzTPN‐Ph) with electron D–A‐based architectures to fabricate nanorods/nanoparticles (FONs) with blue, green, and orange‐red emissions for both one‐photon and two‐photon‐activated fluorescence imaging (Figure 7f,g). Interestingly, these similar structure‐based TADF nanoprobes displayed different morphologies and optical properties, which could be attributed to the different D–A dipole–dipole supramolecular interaction caused by their divergent molecule architectures.^[^ ^29^ ^]^ Furthermore, to improve the specific accumulation of TADF probes in cancer cells, Liu et al. have recently proposed the TADF dyes‐encapsulated liposomes with the modification of galactose ligand, applying as a malignant cell‐targeting fluorescent probe.^[^ ^74^ ^]^

### 4.2 Time‐Resolved Luminescence Imaging and Biosensing

#### 4.2.1 Time‐Resolved Luminescence Imaging (TRLI)

**Figure 8:**
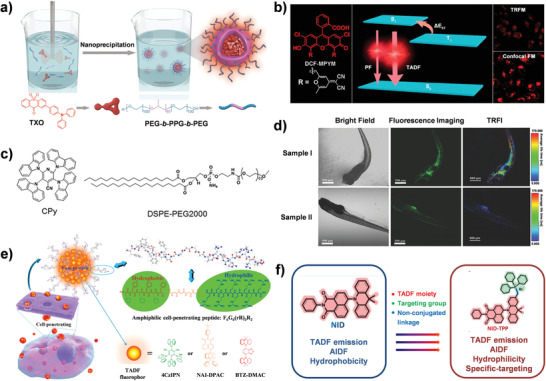
TADF materials for LLIM and TRLI. a) Fabrication of TXO NPs by nanoprecipitation for TP‐FLIM. Reproduced with permission.^[^ ^73^ ^]^ Copyright 2018, WILEY‐VCH. b) TADF of fluorescein derivative DCF‐MPYM for TRLI. Reproduced with permission.^[^ ^23^ ^]^ Copyright 2014, American Chemical Society. c) Structures of CPy and DSPE‐PEG2000. d) Fluorescence images (left and middle) and TRLI (right) of zebrafish. (Sample I/II: injected/non‐injected with CPy‐Odots). c,d) Reproduced under the terms of the Creative Commons CC‐BY license.^[^ ^72^ ^]^ Copyright 2017, The Authors. Published by WILEY‐VCH. e) Cell‐penetrating TADF NPs were prepared via self‐assembly with F_6_G_6_(rR)_3_R_2_. Reproduced with permission.^[^ ^27^ ^]^ Copyright 2018, American Chemical Society. f) The NID‐TPP for TRLI. Reproduced under the terms of the Creative Commons CC‐BY license.^[^ ^69^ ^]^ Copyright 2019, The Authors. Published by WILEY‐VCH.

The luminescence imaging based on intensity realized the visualization of biological activities and pathophysiological processes in cells or subcellular organelles.^[^ ^94^, ^95^, ^96^, ^97^, ^98^ ^]^ However, accuracy and precision of conventional luminescence imaging not only are impeded by the probe concentrations and excitation laser power, but also suffer from the indistinguishable autofluorescence backgrounds in organisms.^[^ ^54^, ^55^, ^99^, ^100^, ^101^, ^102^, ^103^, ^104^ ^]^ Noteworthily, luminescence lifetime imaging (LLIM), by means of the luminescence lifetime instead of intensity as another optical property to be measured, could distinguish the probe signal from background interference in different time domain even in similar emission wavelengths, which has aroused growing interests for bioimaging and biosensing over the past few years.^[^ ^56^, ^105^, ^106^, ^107^, ^108^, ^109^ ^]^ Compared with the common LLIM agents such as lanthanides,^[^ ^54^, ^110^, ^111^ ^]^ transition‐metal complexes,^[^ ^112^, ^113^ ^]^ and inorganic NPs,^[^ ^114^, ^115^ ^]^ which generally exhibit potential heavy metal‐induced cytotoxicity, undesirable degradation, and being expensive‐to‐manufacture, the TADF fluorophores as typical metal‐free organic probes demonstrate excellent biocompatibility and low production cost for LLIM.^[^ ^30^, ^43^, ^53^, ^116^ ^]^ For example, Fan et al. designed a water‐soluble organic semiconducting NPs (TXO NPs) simultaneously combining both TADF and AIE features through encapsulating the thioxanthone‐based TADF fluorophore (TXO) into an amphiphilic polymer matrix (PEG‐*b*‐PPG‐*b*‐PEG) (**Figure** **8**a). Interestingly, the D–A structure of TXO rendered the as‐prepared NPs as efficient candidates for two‐photon fluorescence lifetime imaging (TP‐FLIM). Meanwhile, the oxygen‐inert TXO NPs exhibited a remarkably long luminescence lifetime up to 4.2 µs, which demonstrated promising potential for high‐precision TP‐FLIM in oxygen‐rich biological environments.^[^ ^73^ ^]^

To further improve the specificity and sensitivity, TRLI has been developed to renew the visual diagnosis and sensing based on luminescence lifetime. This imaging technique could completely scavenge the short‐lived background signals in biological environments for enhancing the signal‐to‐noise ratios through setting an appropriate time gating between the excitation laser and detection of the luminescence to only collect the long‐lived signals.^[^ ^42^, ^56^, ^94^ ^]^ To date, TADF dyes have become one of the most attractive materials in TRLI.^[^ ^30^, ^66^, ^67^, ^70^, ^75^, ^76^, ^117^ ^]^ As indicated in Figure 8b, Peng's group proposed a fluorescein derivative DCF‐MPYM with distinctive TADF characteristics, which displayed a long fluorescence lifetime up to 22.11 µs in deaerated ethanol with a narrow Δ*E*_ST_ ≈28.36 meV, showing excellent potential in TRLI of living cells.^[^ ^23^ ^]^ Whereas, the oxygen‐sensitivity of DCF‐MPYM impeded its further bio‐applications. To ameliorate this dilemma, a strategy of isolating the aggregates of TADF molecules (2,3,5,6‐tetracarbazole‐4‐cyano‐pyridine, CPy) with amphiphilic polymers DSPE‐PEG2000 matrix from the oxygen environment was reported by Huang et al. (Figure 8c). The as‐constructed CPy‐based organic dots (CPy‐Odots) demonstrated bright fluorescence (PLQY of 38.3%) and a long lifetime of 9.3 µs in the ambient atmosphere. Additionally, the fluorescence imaging and TRLI in living zebrafish confirmed the CPy‐Odots could be used as a delicate microangiography probe (Figure 8d).^[^ ^72^ ^]^

On the other hand, cell membranes as physiological barriers usually prevent the uptake of the exogenous luminescence agents,^[^ ^118^, ^119^ ^]^ which would delay the imaging in real time. On account of this consideration, Zhao's group designed highly permeable TADF NPs through self‐assembling TADF molecules such as 4CzIPN, NAI‐DPAC, and BTZ‐DMAC with an amphiphilic cell‐penetrating peptide, F_6_G_6_(rR)_3_R_2_, which was based on 20 amino acid residues, showing outstanding biosafety and high cytomembrane permeability (Figure 8e).^[^ ^27^, ^120^ ^]^ The simple and straightforward noncovalent modification method supplied a versatile strategy to fabricate aqueous dispersed TADF NPs with excellent cell permeability for TRLI of living cells in oxygenic environments. As described previously, apart from encapsulating TADF fluorophores within NPs to reduce their intrinsic hydrophobicity in biomedicine, the precise modification of TADF molecular structures would also be applied to alleviate the technological bottleneck caused by poor water solubility. Yang et al. reported a hydrophilic TADF molecule (NID‐TPP), which was fabricated by incorporating a mitochondria‐targeting triphenylphosphonium (TPP^+^) group to NID‐based TADF luminophore (Figure 8f). With the feature of aggregation‐induced delayed fluorescence enhancement, the NID‐TPP displayed obvious red‐emission TRLI in living cells as well as accurate mitochondrial imaging.^[^ ^69^ ^]^

**Figure 9:**
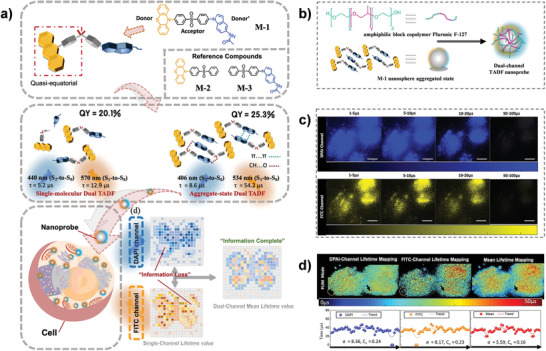
Single‐luminophore dual TADF for TRLI. a) The chemical structure, dual TADF emission in different states, and TRLI effects of M‐1. b) The pretreatment of dual‐channel TADF NPs. c) TRLI in DAPI and FITC channels. d) Integrating TRLI information. Scale bar: 20 µm. Reproduced with permission.^[^ ^12^ ^]^ Copyright 2020, Wiley‐VCH.

Although TADF probes have received justified acclaim from the bioimaging community, the majority of them present only one emission signal, which might cause a loss of biological informational integrality in some complicated microenvironments concurrently with the compromised fluorescence intensity. To overcome this limitation, Zhu et al. developed a single‐luminophore dual TADF (M‐1) based on unsymmetrical donor/acceptor alternate (D–A–D′), displaying a complementarity dual TADF‐band characteristic for the effective alleviation of distortion in TRLI (**Figure** **9**a).^[^ ^12^ ^]^ The dual‐TADF could be achieved not only in monomeric solution, but also in an aggregated state, so trapping M1 within the amphiphilic block copolymer Pluronic F‐127 and formed into nanoprobes could be used to mitigate oxygen quenching for TRLI (Figure 9b). As revealed in Figure 9c, HeLa cells endocytosed with the nanoprobe elaborated dual‐channel (the DAPI and FITC channel) emissions, whose average photoluminescence lifetime in cell were 33 and 36 µs, respectively, realizing the different signal intensity via changing the TRLI parameter. More importantly, outperforming the single‐channel mapping, the dual‐channel mean lifetime mapping could further reduce the TRLI distortion by 30–40% (Figure 9d), opening new horizons of TADF materials for applications in precision medicine.

**Figure 10:**
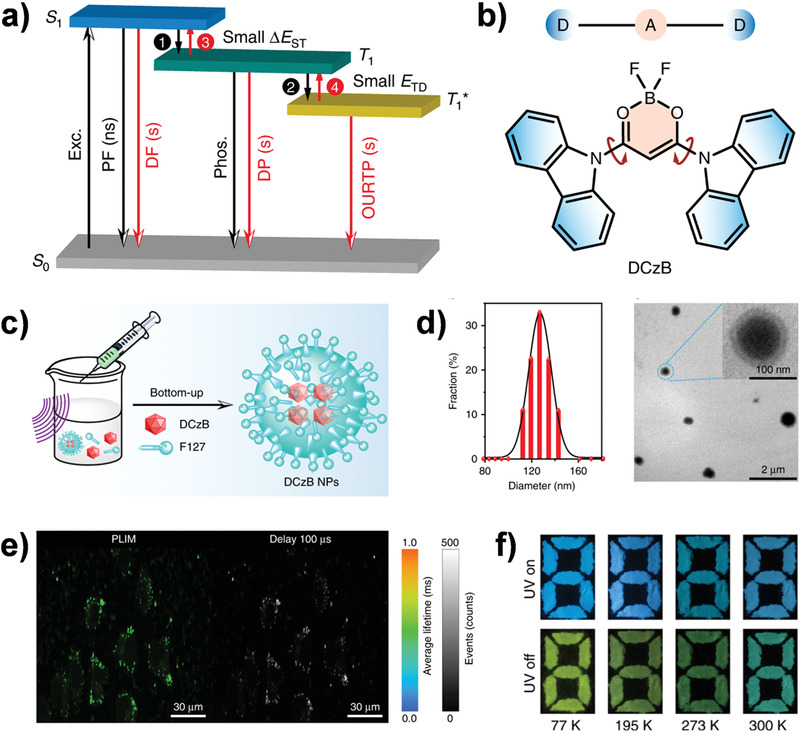
Applications of DCzB NPs for cell imaging and visual temperature detection. a) TAA emission based on TAER (step 4) and RISC processes (step 3). b) The twisted structure of DCzB. c) Preparation of DCzB NPs. d) Dynamic light scattering (DLS) analysis and TEM images of DCzB NPs. e) PLIM and TRLI of cells treated with DCzB NPs. f) Photographs of the DCzB powder before and after removal of the 365 nm UV lamp at 77, 195, 273, and 300 K. Reproduced with permission.^[^ ^13^ ^]^ Copyright 2020, Nature Publishing Group.

Very recently, Huang's group introduced the emission nature of organic afterglow into the TADF progress, achieving exceptionally high afterglow efficiency up to 45%, which enriched the photophysical characteristics of TADF molecules in biomedical applications.^[^ ^13^ ^]^ As elaborated in **Figure** **10**a,b, through bonding difluoroboron *β*‐diketonate with carbazole to construct a twisted D–A architecture of DCzB, a tri‐mode organic afterglow base on thermally activated afterglow (TAA) mechanism could be realized, including: 1) the organic ultralong room temperature phosphorescence (OURTP) from T_1_*; 2) T_1_* excitons transformed to T_1_ via the thermally activated exciton release (TAER) for delayed phosphorescence (DP) due to a shallow trapping depth (*E*_TD_); and 3) S_1_ trapped excitons from T_1_ for DP by RISC processes with a small Δ*E*_ST._ Furthermore, the water‐dispersible DCzB NPs were fabricated by encapsulating DCzB molecules within amphiphilic phospholipid (F127) (Figure 10c,d). Both phosphorescence lifetime imaging (PLIM) and TRLI confirmed that the heavy atom‐free DCzB NPs could be served as promising candidates for high‐precision cell imaging with a long‐lived lifetime of ≈500 µs (Figure 10e). More interestingly, in light of the temperature‐dependent afterglow color, the DCzB afterglow molecules were also explored for visual temperature detection. As shown in Figure 10f, different from DCzB powder under UV light excitation showing a neglectable color change from 300 to 77 K, the DCzB crystals displayed a blue‐green to green‐yellow afterglow color variation after turning off the UV light.

#### 4.2.2 Time‐Resolved Fluorescence Biosensing

**Figure 11:**
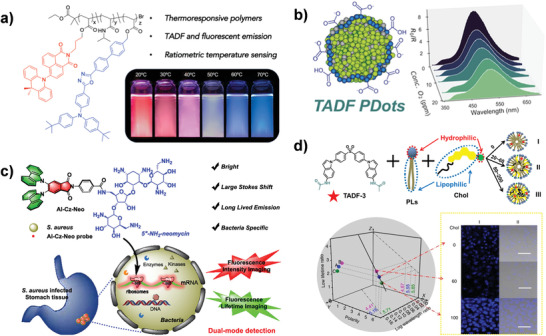
TADF materials for time‐resolved fluorescence biosensing. a) Illustration of NAI‐based TADF polymers for ratiometric temperature sensing. Reproduced with permission.^[^ ^71^ ^]^ Copyright 2020, American Chemical Society. b) Scheme of TADF PDots (green: PTZ‐ODA_0.15_, blue: PSMA), and the emission intensity at 516 nm scaled to its at 396 nm with oxygen concentrations variation of PTZ‐ODA_0.15_ in PDots. Reproduced with permission.^[^ ^32^ ^]^ Copyright 2020, American Chemical Society. c) Schematic illustration of the TADF probe (AI−Cz−Neo) for dual‐mode detection of bacterial 16S rRNA in tissues. Reproduced with permission.^[^ ^15^ ^]^ Copyright 2020, American Chemical Society. d) The fabrication of TADF‐3‐based membranes nanostructures with different Chol contents, and its 3D sensing application in simulated membranes. Reproduced with permission.^[^ ^28^ ^]^ Copyright 2019, Nature Publishing Group.

In view of the sensitivity of RISC processes to thermal energy and molecule oxygen, the TADF materials hold great promise for both temperature and oxygen sensing.^[^ ^31^, ^32^, ^57^, ^71^ ^]^ As shown in **Figure** **11**a, Hudson and co‐workers first synthesized TADF monomer based on naphthalimide (NAI) acceptor and dimethylacridine (DMAC) donor, exhibiting orange to deep‐red TADF emission. Subsequently, copolymerizing the NAI‐DMAC monomer with a blue fluorophore (*t*BuODA) and *N*‐isopropylacrylamide (NIPAM) matrix to construct thermo‐responsive polymers, which were used as ratiometric temperature sensors with the variation from red TADF emission at room temperature to blue fluorescence at 70 °C, achieving dual‐detection of temperature based on visible color and lifetime change.^[^ ^71^ ^]^ Furthermore, Hudson et. al. also designed the TADF material (PTZ‐ODA_0.15_) copolymerized of phenothiazine/oxadiazole donor/acceptor monomers (TPZ‐ODA) and a carbazole‐based host (CzBA), which showed ratiometric O_2_ concentration‐related dual emission of prompt and delayed fluorescence peaks. To improve its application in biological systems, the PTZ‐ODA_0.15_ was coprecipitated with amphiphilic polystyrene/maleic anhydride copolymer (PSMA) into water‐soluble polymer dots (PDots) for ratiometric oxygen sensoring (Figure 11b).^[^ ^32^ ^]^

Besides oxygen, the TADF materials could also be developed as sensors for the detection of hypochlorite,^[^ ^121^ ^]^ sulfite,^[^ ^122^ ^]^ ribosomal RNA (rRNA),^[^ ^15^ ^]^ cysteine,^[^ ^123^ ^]^ BSA,^[^ ^124^ ^]^ and microenvironmental polarity change.^[^ ^28^ ^]^ For instance, based on TADF property, a fluorescence probe (AI‐Cz‐Neo) was developed by conjugating a TADF molecule with a bacterial 16S rRNA‐targeting neomycin for the fluorescence intensity imaging and TRLI‐based dual‐mode detection of 16S rRNA in cells and tissues (Figure 11c).^[^ ^15^ ^]^ Recently, a 3D ratiometrically luminescent sensor based on a D–A–D TADF molecule (TADF‐3) simultaneously emitting fluorescence and TADF has been reported by Zhu and co‐workers, in which both wavelength and lifetime of the TADF are related to environmental polarity while the fluorescence keeps unchanged towards polarity variation. To explore the potential of such 3D sensors in practical precise diagnosis, various simulated membranes were established by self‐assembling two fundamental membrane components phospholipids (PLs) and cholesterol (Chol) with TADF‐3 molecules into micelle nanostructures, displaying different polarity because of different Chol contents. As expected, luminescent signals of the TADF‐3 molecules in the as‐assembled micelle nanostructures could be utilized to quantitatively detect the polarity variation via a 3D plot diagram (Figure 11d). More attractively, the accurate sensing strategy based on TADF has also been verified in cellular levels, which holds great promise for the detection of cholesterol‐related membrane lesions.^[^ ^28^ ^]^

### 4.3 ^1^O_2_ Generation for PDT

Photodynamic therapy (PDT) with their unique merits of non‐invasion, high specificity, minimal drug resistance, and low side effects, has been approved for clinical utilization in 1993, emerging as one of the most promising modalities for cancer therapeutics.^[^ ^125^, ^126^, ^127^, ^128^, ^129^, ^130^, ^131^, ^132^ ^]^ In PDT, the photosensitizers (PSs) are excited by photons to transfer energy to the surrounding ground state triplet oxygen (^3^O_2_) for the formation of singlet oxygen (^1^O_2_), which will damage tumor cells.^[^ ^58^, ^59^, ^133^, ^134^ ^]^ As indicated above, the photophysical mechanism of TADF materials in PDT elaborates that an effectual ISC process is highly essential for ^1^O_2_ generation.^[^ ^135^, ^136^, ^137^ ^]^ Different from the conventional procedure of introducing heavy‐metal atoms into PSs to improve the ISC process which could potentially induce intrinsic toxicity and undesirable degradation concerns,^[^ ^133^, ^138^, ^139^, ^140^, ^141^ ^]^ the TADF emitters as one of purely organic PSs have aroused growing interest in PDT owing to its fascinating properties especially the metal‐free high‐performance photosensitizing ability.^[^ ^142^, ^143^, ^144^, ^145^, ^146^ ^]^

**Figure 12:**
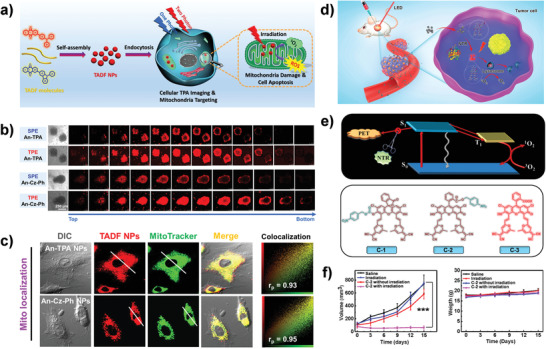
TADF materials for ^1^O_2_ generation in PDT. a) Scheme of the assembled TADF NPs for two‐photon excited fluorescence imaging and mitochondria‐targeted PDT. b) The SPE and TPE penetration depth images of the An‐TPA NPs and An‐Cz‐Ph NPs‐treated A549 multicellular tumor spheroids (MCTSs). c) Mitochondria colocalizations of the A549 cells treated with the An‐TPA NPs and the An‐Cz‐Ph NPs. a‐c) Reproduced with permission.^[^ ^14^ ^]^ Copyright 2019, American Chemical Society. d) Schematic illustration of the NTR‐activatable TADF fluorescein derivatives for turn‐on fluorescence imaging and efficient PDT under mild hypoxia. e) The design strategy of the TADF theranostic molecule and molecular structures of C‐1, C‐2, C‐3. f) PDT efficiency of C‐2 in mice. d,s) Reproduced with permission.^[^ ^142^ ^]^ Copyright 2019, American Chemical Society.

As proof of concept, for the first time, our group proposed water‐dispersible TADF NPs with small Δ*E*_ST_ as a novel metal‐free organic PS for photo‐excited ^1^O_2_ formation in 2016.^[^ ^25^ ^]^ Considering the limitations of unsatisfactory ^1^O_2_ quantum yield, poor optical penetration depth, and lack of organelle targeting, we further designed the two‐photon‐excited (TPE) anthraquinone derivatives‐based TADF NPs (An‐TPA NPs and An‐Cz‐Ph NPs) with favorable ^1^O_2_ quantum yield up to 52% as well as inherently mitochondria targeting capability (**Figure** **12**a).^[^ ^14^ ^]^ It has been well proved that the mitochondria are of vital importance in energy supply and cell apoptosis, being considered as an ideal sub‐cellular target for PDT because of its high vulnerability towards ROS.^[^ ^147^, ^148^, ^149^, ^150^, ^151^, ^152^ ^]^ As described in Figure 12b, TPE images of the TADF NPs‐treated A549 3D multicellular tumor spheroids (MCTSs) revealed a superior near‐infrared (NIR) light penetration depth when compared to the single‐photon‐excited (SPE) images. Furthermore, the as‐prepared TADF NPs displayed intrinsic cancer‐mitochondria‐targeting abilities, which are beneficial to the final PDT performance in cancer treatments (Figure 12c). Apart from mitochondria, the lysosomes have also been regarded as a highly potential target to amplify the PDT efficacy, because ROS produced by PDT would disrupt the lysosomes resulting in extensive acid hydrolases release and subsequently induces cancer cells' programmed death.^[^ ^33^, ^142^, ^153^ ^]^ As depicted in Figure 12d,e, Peng's group developed two TADF fluorescein derivatives (C‐1, C‐2) for nitroreductase (NTR)‐activated fluorescence and PDT. The resultant lysosomes‐targeting TADF compounds could suppress the competitive photoinduced electron transfer, realizing a much higher PDT efficiency than that of the traditional porphyrin (PpIX) even under mild hypoxia conditions.^[^ ^142^ ^]^ Without the NTR inhibition by dicoumarin, the C‐2 could transform to C‐3 via an enzymatic cleavage reaction catalyzed by NTR, turning on the fluorescence in tumor. Moreover, the excellent PDT performance and great biosafety of C‐2 in HeLa tumor‐bearing mice have also been proved (Figure 12f). Recently, to further improve the performance of TADF materials in PDT, Lee's group introduced heavy‐atom effect into the TADF molecule (AQCz) to construct AQCzBr_2_ PSs, which displayed the boosted spin–orbit coupling (SOC) to increase the ISC progress, leading to a considerably high ^1^O_2_ quantum yield (91%).^[^ ^154^ ^]^

**Figure 13:**
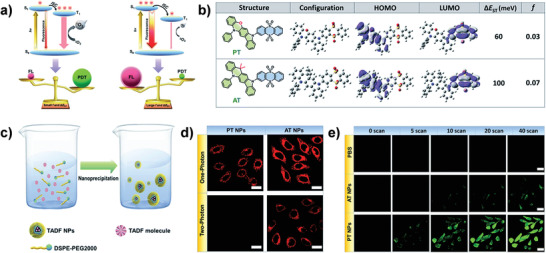
Manipulating exciton dynamics of TADF nanotheranostics. a) Exciton dynamics in TADF molecules. b) Molecular structures, configuration, HOMO, LUMO, and the value of Δ*E*_ST_ and *f* of AT and PT. c) TADF NPs preparation. d) SPE and TPE fluorescence images of TADF NPs in cells. e) ^1^O_2_ generation of TADF NPs. Reproduced with permission.^[^ ^16^ ^]^ Copyright 2020, The Royal Society of Chemistry.

Through delicate regulation of the exciton dynamics to tune energy disposition in TADF NPs with D–A structures, the photochemical properties could be manipulated for balancing the fluorescence imaging and PDT. Lee et al. engineered two TADF molecules (PT and AT) with different electron‐donating segments to tailor the Δ*E*_ST_ and oscillator strength (*f*). After preparing these molecules into nanotheranostic agents, the as‐fabricated PT and AT NPs show different capabilities in TPE ^1^O_2_ generation and fluorescent emission (**Figure** **13**a–c).^[^ ^16^ ^]^ As shown in Figure 13d,e, in comparison to AT NPs with stronger intensive fluorescence, the PT NPs were more beneficial for PDT contributed to the more efficient ISC process owing to smaller Δ*E*_ST_ and *f* in PT molecule. These results proposed an effective strategy to design TADF nanotheranostics for highly efficient PDT or high‐performance fluorescence imaging by tailoring the Δ*E*_ST_ and *f* of TADF materials.

## 5 Conclusion

As metal‐free organic materials, TADF materials with unique photophysical properties, tailorable synthesis, and low‐cost manufacture, have demonstrated promising potential in biomedical fields: 1) with the prompt fluorescence as well as the long‐lived delayed fluorescence, TADF agents could be utilized as probes for conventional fluorescence imaging, LLIM, or TRLI; 2) the oxygen and temperature‐sensitive RISC processes can accordingly affect the fluorescence lifetime, thus realizing the application of the TADF agents in time‐resolved sensing; and 3) the small Δ*E*_ST_ and consequent effectual ISC process of the TADF molecules render them superior organic PSs for PDT application. Furthermore, rational design strategies such as molecular engineering, self‐assembly, reprecipitation with polymers, and encapsulation within different nanocarriers, have been explored to expand the various applications of intrinsically hydrophobic TADF dyes in physiological environments.

Although a wave of efforts have been devoted to the exploration of TADF materials in biomedicine, such cutting‐edge metal‐free luminophore is still in the early stage of its development. The challenges and opportunities associated with TADF materials coexist, and several essential issues should be addressed before further broad applications as well as preclinical/clinical translations of TADF materials: 1) to alleviate the inherent properties of poor water solubility and undesired bioavailability, more effective methods for modification should be researched, where the targeting ability and large‐scale production should be highly considered; 2) to achieve state‐of‐the‐art properties such as a prolonged luminescence lifetime, suppressed ACQ and O_2_ quenching, and superior ^1^O_2_ quantum yield, more efforts should be made to explore the structure–activity relationship of TADF materials in detail, facilitating the new design theories and concepts of TADF materials, for example, integration with other materials such as AIEgens, afterglow molecules; 3) to realize the deep‐tissue theranostics and eliminate the side effects to healthy tissues caused by short wavelength excitation, the NIR‐I/II TADF emitters should be designed intentionally, which will also extend the applications beyond two‐photon excitation; moreover, 4) to construct smart or multifunctional TADF‐based theranostic platforms, appropriate procedures to chemically or physically incorporate other functional moieties into TADF materials is necessary; and finally, 5) to further evaluate the long‐term biocompatibility and biosafety of TADF materials for practical transformation, comprehensive biodegradability and body clearance functions of TADF fluorophores should be systematically investigated both in vitro and in vivo, and more related pharmacokinetic parameters such as absorption, distribution, metabolism, and excretion (ADME) as well as the toxicity in human tumors‐simulated animal models (e.g., patient‐derived xenografts, humanized mouse models) should be assessed.

With this review, we hope to attract tremendous attention and broader interest directed toward the design of TADF materials for precision medicine. We also believe that with the multiple disciplines‐joined efforts such as physics, chemistry, material sciences, and biology, more significant advance of TADF materials in biomedical applications from diagnostic imaging, biosensing, and PDT to other fields such as synergistic therapy, regenerative engineering, and other disease treatments will be achieved in the near future.

## Conflict of Interest

The authors declare no conflict of interest.
